# Registry data for cross-country comparisons of migrants' healthcare utilization in the EU: a survey study of availability and content

**DOI:** 10.1186/1472-6963-9-210

**Published:** 2009-11-18

**Authors:** Signe Smith Nielsen, Allan Krasnik, Aldo Rosano

**Affiliations:** 1 University of Copenhagen, Department of Public Health, Section for Health Services Research, Øster Farimagsgade 5, 1014 Copenhagen K, Denmark; 2 Agency of Public Health, Latium Region, Via di Santa Costanza 53, 00198 Rome, Italy

## Abstract

**Background:**

Cross-national comparable data on migrants' use of healthcare services are important to address problems in access to healthcare; to identify high risk groups for prevention efforts; and to evaluate healthcare systems comparatively. Some of the main obstacles limiting analyses of health care utilization are lack of sufficient coverage and availability of reliable and valid healthcare data which includes information allowing for identification of migrants. The objective of this paper was to reveal which registry data on healthcare utilization were available in the EU countries in which migrants can be identified; and to determine to what extent data were comparable between the EU countries.

**Methods:**

A questionnaire survey on availability of healthcare utilization registries in which migrants can be identified was carried out among all national statistic agencies and other relevant national health authorities in the 27 EU countries in 2008-9 as part of the Migrant and Ethnic Minority Health Observatory-project (MEHO). The information received was compared with information from a general survey on availability of survey and registry data on migrants conducted by Agency of Public Health, Lazio Region, Italy within the MEHO-project; thus, the information on registries was double-checked to assure accuracy and verification.

**Results:**

Available registry data on healthcare utilization which allow for identification on migrants on a national/regional basis were only reported in 11 EU countries: Austria, Belgium, Denmark, Finland, Greece, Italy, Luxembourg, the Netherlands, Poland, Slovenia, and Sweden. Data on hospital care, including surgical procedures, were most frequently available whereas only few countries had data on care outside the hospital. Regarding identification of migrants, five countries reported having information on both citizenship and country of birth, one reported availability of information on country of birth, and five countries reported availability of information on citizenship.

**Conclusion:**

Lack of registry data in 16 EU countries, shortage of data on healthcare utilization, and the diversity in the definition of migrant status hampers cross-national comparisons and calls for an urgent establishment of registries, expansion of the existing registry information, and adoption of a common, generally acceptable definition and identification method of migrants across the EU.

## Background

Migrant health and access to healthcare are fundamental elements of integration and human rights. Most EU countries grant equal access to healthcare for migrants with permanent residence compared to non-migrants [1,2]; nevertheless, differences in health care utilization between migrants and the indigenous European populations have been documented [3-7]. This could reflect inequality in terms of problems in accessibility to health care services due to informal barriers such as language difficulties, cultural differences, trauma, and newness [8-11]>, or they could reflect differences in health needs. A first step to address these issues from a public health perspective is to monitor migrants' use of healthcare services. Likewise, data on healthcare use are helpful in identifying high risk groups; hence, a better basis for prevention efforts targeting the individual migrant groups will be created. This requires reliable and valid data on healthcare utilization which includes information allowing for identification of migrant groups in the EU countries.

Furthermore, cross-country comparisons of various migrant groups living in different EU countries could provide us with a more comprehensive picture of migrant health and issues concerning the health systems. Internationally comparable data can also be employed to evaluate healthcare systems against those of other countries.

In this paper, we only focus on registry data as registries can contain information on the entire population, are often more accurate than self-report [12-15], and have regularly updated information. Surveys, on the contrary, are often only available in the national language, and migrants often have low response rates in these surveys due to language difficulties and a general lack of trust and contact to the surrounding society as well as due to less positive experiences as receivers of general inquiries from official institutions in the receiving country and in their homeland [16]. Thus, the sample is not representative for the migrant population. Also, surveys are often not carried out regularly.

To our knowledge, no overview of migrant-specific registry data on healthcare utilization in the EU countries exists. Therefore, the objective of this paper was to identify 1) which registry data on healthcare utilization were available in the EU countries in which migrants can be identified; 2) to what extent data were comparable between the EU countries. The work is part of the Migrant and Ethnic Health Observatory (MEHO)-project funded by the European Commission. The major objectives of the project are to construct an inventory of existing data sources on migrant health across EU member states and to develop migrant and ethnic-specific indicators within different areas, including health care utilization.

## Methods

As no universally agreed on operational definitions currently exist for categorising migrants, the following definition developed by the MEHO-partners was employed: "A migrant is any person who migrated to the current EU-27 countries from outside the EU-15 member states (the 15 EU member states before the expansion in 2004), while further excluding North America and Australasia but including the post World War II guest workers from the Southern European countries periphery (e.g. Italy, Greece, and Turkey) and re-settlers from the former countries of the Soviet Union, and is staying as a resident (not a visitor, asylum seeker, temporary worker or student)" [17]. Thus, large group of migrant workers from countries like Italy and Greece are included in the definition in spite of the long lasting EU membership of their country of origin. This is primarily motivated by the expectation of the MEHO partners that these migrant groups might be disadvantaged healthwise and therefore should be included in the various parts of the MEHO project. Furthermore, by using this definition, ethnic minorities living in a country for generations were excluded.

For the purpose of gaining knowledge about existing registries on health care utilization which includes information allowing for identification of permanently settled migrants in the EU countries, we designed a questionnaire. Due to national differences in registry and classification systems, we pilot tested the questionnaire among our collaboration partners, mainly researchers and national health authorities, in selected EU countries (Denmark, Germany, Italy, the Netherlands, and the UK). The questionnaire contained information on healthcare utilization registry sources including background information of registry such as demographic catchment area, number of records, delimitation of data, and registry format; availability of health care utilization information; availability of identification of migrants; availability of demographic and socio-economic information; limitations of data; and access to the registry.

We collected contact information of members of the Statistical Programme Committee network group, coordinated by EUROSTAT, which represents the official channel for statistical information in each EU country to whom we sent the questionnaire by email including a cover letter which explained the objectives in details, and that the research project was part of the MEHO-project [17]. Furthermore, it was specified that accurate and complete information was extremely important for the development of this research project. Finally, it was outlined that if the receiver of the questionnaire did not represent the relevant institution, the receiver was kindly asked to provide us with contact information about who we should contact instead in the country in question.

After the deadline for the reply of the questionnaire specified in the cover letter, a reminder was sent along with the questionnaire. This procedure was repeated three times for countries not responding. Nevertheless, after four reminders, Italy, Malta, Spain, and the UK still did not respond (or did not respond with complete information). For Italy and Malta, we received the information on registries on healthcare utilization from another survey on general information on registries and survey data on migrant health that was conducted by Agency of Public Health, Lazio Legion, Italy in 2007/8 within the MEHO-project. Furthermore, we contacted researchers in Spain and the UK with expertise within healthcare utilization and migrant health with whom we had a personal contact. Through our contacts in Spain, the Health Ministry of Spain provided us with the information regarding national registries in Spain. For the UK, we only received information through our personal contacts; however, the different informants from the UK all concluded the same regarding availability of the registries. Finally, we double-checked all our collected information on healthcare utilization registries with information from the general survey of 2007/8. If a mismatch of the information occurred (in three cases: Austria, Belgium, and Czech Republic), we contacted the respondents for a clarification. All information on the registries was collected from September 2008-May 2009.

Criteria for registries to be listed in this overview were: 1) information on utilization of at least one healthcare service should be covered including a) in-patient hospital care utilization such as hospital admission and length of stay; b) out-patient care utilization such as ambulatory contacts, emergency room contacts, and contact to general practitioners; c) surgical operation and procedures; and d) medicine use; 2) information on migrant status should be included by at least one of the following indicators either directly or by linkage: country of birth, mother's country of birth, father's country of birth, citizenship, mother's citizenship, or father's citizenship according to the MEHO-definition; 3) the registry should have preferably national coverage or at least regional coverage; 4) the registry should include the general population and not a population sub-group e.g. infants, children, etc.

Registries on healthcare use for specific diagnoses or conditions e.g. reproductive health were excluded in order to generate a general overview.

## Results

An overview of available registries on migrant-specific healthcare utilization in the EU countries is provided in table 1 and 2. From the tables, it can be derived that we were able to collect information on registry data as defined in 11 countries; namely Austria, Belgium, Denmark, Finland, Greece, Italy, Luxembourg, the Netherlands, Poland, Slovenia, and Sweden. Hence, 16 countries (Bulgaria, Cyprus, Czech Republic, Estonia, France, Germany, Hungary, Ireland, Latvia, Lithuania, Malta, Portugal, Romania, Slovakia, Spain, and the UK) reported to have no available registries on healthcare utilization in which migrants could be identified. However, for Spain, it was not possible for us to obtain contact with all 17 regional health authorities for which reason we can only rule out the existence of national registries but not completely the existence of regional registries.

**Table 1:** Available registries on healthcare utilization by EU country

**Country and Name of registry**	**Availability of healthcare utilization data**^**1**^													
	**In-patient hospital care utilization**					**Out-patient care utilization**					**Surgical operations**			**Prescribed medicine**
	**All hospital admission**	**Acute care hospital admission**	**Length of stay (all)**	**Length of stay (acute)**	**All outpatient contacts**	**Hospital day cases**	**ER**	**GP**	**Medical Specialist**	**Dentists**	**All procedures (in-patients + day cases)**	**Surgical in-patients**	**Surgical daycases**	**Purchases of prescribed medicine**
**Austria**														
Hospital Discharges	X	X	X	X							X	X	X	
**Belgium**														
Minimal Clinical Data	X		X			X					X^2^			
**Denmark**														
National Patient Registry	X	X	X	X	X	X	X				X	X	X	
National Health Insurance Registry								X	X	X			X^3^	
Medicine Registry														X
**Finland**														X
Hospital Discharge Registry	X		X			X					X	X	X	
Registry on Outpatient Visits in Public Hospitals						X^4^								
National Health Insurance Registry									X^5^					X^6^
**Greece**														
Survey on Discharged Hospital Patients	X	X	X	X							X	X		

All registries have national coverage.

^1^Including mental health care and rehabilitation but excluding nursery home care.

^2 ^The registry contains this piece of information; however, only for certain/specific illness/diagnoses/condition.

^3 ^Only for specialist doctors.

^4 ^Only consultation of medical specialists in public hospitals.

^5 ^Only reimbursed use of private healthcare services.

^6 ^Only reimbursed purchases of prescribed medicine.

Limitation of data: *Austria*, *Hospital discharges*: A personal ID is not available, data represent single cases of discharges including repeated discharges of the same person. *Finland, Registry on Outpatient Visits in Public Hospitals*: Private services excluded. *National Health Insurance Registry*: Only reimbursed medicine and private healthcare services are included. *Greece, Survey on Discharged Hospital Patients*: A monthly survey is conducted among all patients who get hospitalised in Greece. The National Statistical Service of Greece gather the information stemming from the surveys.

**Table 2:** Available registries on healthcare utilization by EU country

**Country and Name of registry**	**Availability of healthcare utilization data**^**1**^													
	**In-patient hospital care utilization**					**Out-patient care utilization**					**Surgical operations**			**Prescribed medicine**
	**All hospital admission**	**Acute care hospital admission**	**Length of stay (all)**	**Length of stay (acute)**	**All outpatient contacts**	**Hospital day cases**	**ER**	**GP**	**Medical Specialist**	**Dentists**	**All procedures (in-patients + day cases)**	**Surgical in-patients**	**Surgical daycases**	**Purchases of prescribed medicine**
**Italy**														
Hospital Information System	X	X				X	X				X	X	X	
Sistema Informativo Emergenza Sanitaria							X							
Sias (Sistema Informativo Assistenza Specialistica)									X					
**Luxembourg**														
Hospital Registry	X										X	X		
**Netherlands**														
Hospital Discharge Registry (HDR)	X	X	X	X		X					X	X	X	
Netherlands Information Network of General Practice								X						
**Poland**														
Hospital Discharge Form	X		X	X		X					X	X	X	
**Slovenia**														
Database on Hospital Treatments	X	X				X	X				X			
**Sweden**														
National Patient Discharge Registry	X					X	X				X			
The Swedish Prescribed Drug Registry														X

All registries have national coverage except for Sistema Informativo Emergenza Sanitaria (Italy) which only covers three-four regions.

^1^Including mental health care and rehabilitation but excluding nursery home care.

Limitation of data: *Netherlands, Hospital Discharge Registry (HDR)*: From 2006 onwards, HDR has limited coverage of hospitals (non-response of 10-13% of discharges), which affects the linkage. From 2005/2006 ca. 30% of the hospitals do not register procedures; so these are estimated.

Note: Estonia has targeted to launch digital health record system by 2012 which will enable Estonia to start analyses that base on individual data, including personal characteristics.

Greek data stemmed from a monthly survey carried out among all hospitalized patients which fed into an electronic database. This data record could function as a registry and was therefore included in this paper.

### Hospital care

In all 11 countries, information on hospital utilization was available whereas information on length of stay in the hospital was only available in eight countries. Specific data on acute hospital admission was less frequently available, seven countries collected data on this, and five countries had further information on the length of stay for acute hospitalization.

Information on hospital day cases were available in eight countries (Belgium, Denmark, Finland, Italy, the Netherlands, Poland, Slovenia, and Sweden), and emergency room contacts were registered in four countries (Denmark, Italy, Slovenia, and Sweden).

Data on surgical procedures were available in all 11 countries while more detailed information on this matter (in-patients or ambulatory patients) was available in eight and six countries, respectively.

### Care outside hospital

Care outside hospital was less frequently available in the registries. Consultation of general practitioners (GP) was registered in only Denmark and the Netherlands, consultation of medical specialists (outside the hospital) was registered in Denmark, Finland, and Italy, and consultation of dentists was only registered in Denmark.

### Prescribed medicine purchase

Purchases of prescribed medicine were only registered in the Scandinavian countries: Denmark, Finland, and Sweden.

### Indicators of migrant status

**Figure 1:**
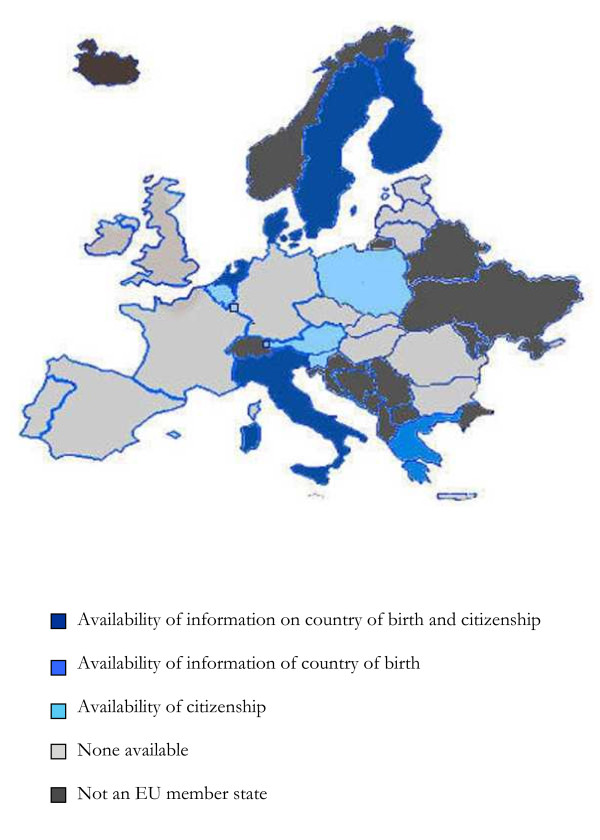
**Availability of registry information allowing for identification of migrants in the EU**.

Identification of persons with migrant background in the registry data relied on a) country of birth (and in some cases, additional information of parents), and/or b) citizenship (and in some cases, additional information of parents) (table 3). In Austria, Belgium, Luxembourg, Poland, and Slovenia only information on citizenship was available, in Greece only information on country of birth was available, and in Denmark, Finland, Italy, the Netherlands and Sweden both information on citizenship and country of birth was available; and additionally, in Denmark, the Netherlands, and Sweden this information was also available for the parents (figure 1).

**Table 3:** Available registry information of migrant indicators and socio-economic status by EU country

**Country**	**Availability of migrant indicator**						**Socioeconomic status**	**Limitations of data**
	**Country of birth**	**Mother's country of birth**	**Father's country of birth**	**Citizenship**	**Mother's citizenship**	**Father's citizenship**		
**Austria**				D			Age, sex (D)	
**Belgium**				D			Age, sex (D)	Citizenship: only options are Belgium, EU, the rest of the world
**Denmark**	L	L	L	L	L	L	Age, sex and all major socio- economic indicators (L)	Information on mothers'/fathers' citizenship and country of birth is only available if the mother/father is or has been resident in Denmark
**Finland**	L			L			Age, sex and all major socio- economic indicators (L)	
**Greece**	L						Age, sex and all major socio- economic indicators (L)	
**Italy**	D			D	L*	L*	Age, sex, marital status, and for some regions education (the latter not for Sias) (D)	Country of birth is problematic variable as many ethnic Italians are born in e.g. South America and Africa. Citizenship data are not of high quality in all regions (not collected correctly due to coding issues)
**Luxembourg**				D			Age, sex (D)	
**Netherlands**	L	L	L	L	L	L	Age, sex and all major socio- economic indicators (L)	Missing discharges cannot persons individually be linked to and are not evenly distributed among migrant groups
**Poland**				L			Age, sex (L)	
**Slovenia**				D			Age, sex and all major socio- economic indicators (D)	Data on citizenship are not realistic before 2006
**Sweden**	L	L	L	L	L	L	Age, sex and all major socio- economic indicators (L)	Population registries of residents include a considerable number of foreign-born that have their main residence outside of Sweden. The magnitude of this problem is not well defined.

D = Directly available in the Registry

L = Available by linkage

* For persons born after 1990

Nonetheless, quality of the data on migrant status is essential. Limited information on citizenship was available in Belgium with only three options (Belgium, EU, and the rest of the world). In Denmark, information on parents' citizenship and country of birth was only available if the parents were or had been resident in Denmark. In Italy, data on citizenship was not always collected correctly due to coding issues, and thus, this indicator was not reliable. In the Netherlands, linkage failure was high among some elderly immigrant groups (especially from Moroccan origin) due to unknown dates of birth, and in Sweden, the population registries of residents included a considerable number of foreign-born that had their main residence outside of Sweden; the magnitude of this problem was not well defined.

### Indicators of demographic and socio-economic status

Age and gender but also socio-economic variables are important determinants of healthcare utilization [18,19]; hence, useful for registry and analyses purposes. The registries in all 11 countries had at least age and gender information (table 3) whereas Denmark, Finland, Greece, the Netherlands, Slovenia and Sweden had additional registry-based information on socio-economic variables. For Italy, socio-economic information (education) was only available in some regions.

### Comparisons possibilities

In table 4, an overview of comparison options by healthcare services and indicators of migrant background is given. Employing citizenship as a migrant background indicator would allow for a comparison between most countries. By citizenship, number of hospital admissions and all surgical procedures can be compared across 10 countries followed by hospital day cases (8 countries), and length of stay and surgical in-patients (7 countries). By the country of birth indicator, number of hospital admissions and all surgical procedures can be compared across 6 countries followed by hospital day cases and surgical in-patients (5 countries), and length of stay, acute care admissions, and surgical day cases (4 countries). Only Denmark seems to have information on number of dentist consultation; consequently, this healthcare service using registry data cannot be compared across countries. In general, the possibilities for cross-national comparisons for all out-patient care indicators (except for hospital day cases) and prescribed medicine are very limited.

**Table 4:** Grouping of EU countries according to availability of data by type of healthcare service and migrant indicator

**Healthcare service**	**Migrant indicator**	**N**	**Countries with availability**
**In-patient hospital care utilization**			
All hospital admissions	Country of birth	6/27	Denmark, Finland, Greece, Italy, the Netherlands, Sweden
	Citizenship	10/27	Austria, Belgium, Denmark, Finland, Italy, Luxembourg, the Netherlands, Poland, Slovenia, Sweden
Acute care hospital admissions	Country of birth	4/27	Denmark, Greece, Italy, the Netherlands
	Citizenship	6/27	Austria, Denmark, Italy, the Netherlands, Poland, Slovenia
Length of stay, all hospitals admissions	Country of birth	4/27	Denmark, Finland, Greece, the Netherlands
	Citizenship	7/27	Austria, Belgium, Denmark, Finland, Luxembourg, the Netherlands, Poland
Length of stay, acute care	Country of birth	3/27	Denmark, Greece, the Netherlands
	Citizenship	4/27	Austria, Denmark, the Netherlands, Poland
**Out-patient care utilization**			
All outpatient (ambulatory) contacts	Country of birth	1/27	Denmark
	Citizenship	1/27	Denmark
Hospital daycases	Country of birth	5/27	Denmark, Finland, Italy, the Netherlands, Sweden
	Citizenship	8/27	Belgium, Denmark, Finland, Italy, the Netherlands, Poland, Slovenia, Sweden
Emergency room contacts	Country of birth	3/27	Denmark, Italy, Sweden
	Citizenship	4/27	Denmark, Italy, Slovenia, Sweden
Consultation of general practitioners	Country of birth	2/27	Denmark, the Netherlands
	Citizenship	2/27	Denmark, the Netherlands,
Consultation of medical specialists	Country of birth	3/27	Denmark, Finland, Italy
	Citizenship	3/27	Denmark, Finland, Italy
Consultation of dentists	Country of birth	1/27	Denmark
	Citizenship	1/27	Denmark
**Surgical operations and procedures**			
All surgical procedures (in-patients + daycases)	Country of birth	6/27	Denmark, Finland, Greece, Italy, the Netherlands, Sweden
	Citizenship	10/27	Austria, Belgium, Denmark, Finland, Italy, Luxembourg, the Netherlands, Poland, Slovenia, Sweden
Surgical in-patients	Country of birth	5/27	Denmark, Finland, Greece, Italy, the Netherlands
	Citizenship	7/27	Austria, Denmark, Finland, Italy, Luxembourg, the Netherlands, Poland
Surgical daycases	Country of birth	4/27	Denmark, Finland, Italy, the Netherlands
	Citizenship	6/27	Austria, Denmark, Finland, Italy, the Netherlands, Poland
**Prescribed medicine purchases**			
Purchases of prescribed medicine	Country of birth	3/27	Denmark, Finland, Sweden
	Citizenship	3/27	Denmark, Finland, Sweden

## Discussion

Our study revealed the existence of registry data on healthcare utilization in 11 EU countries; consequently, there is a significant gap in data availability in the EU. No pattern of the geographical placement of countries with data availability is seen, yet, four of the large countries in EU (Germany, France, Spain, and the UK) did not report available data. Data on in-patient hospital care including surgical procedures are most frequently available whereas only few countries seem to have general registry data on out-patient care and medicine purchases. Regarding identification of migrants in the registries, five countries reported information on citizenship, just one reported having information on country of birth, and five countries information on both citizenship and country of birth.

### Methodological issues

Firstly, the reliability and comprehensiveness of the information on migrant-specific healthcare utilization registries in EU relies on our informants in the different countries. Consequently, lack of knowledge or slipshod work when filling in the questionnaire might lead to missing relevant registries in this study. Although, we have tried to overcome this problem by a double-check of the information by comparing the information with information from the more general survey on availability of survey and registry data on migrants and seeking out the eventually mismatches, there might still be errors. This can especially be true for Spain as we could not obtain information for all 17 regions. Secondly, we only chose to include general healthcare registries; thus, essential information on specific kinds of healthcare use might be available in other major registries e.g. cancer registries in the different countries.

Migrants and ethnic minorities are defined differently in the EU countries [17]; this also affects the information collected in registries. The registry information on this matter is pragmatic, crude, and measures either country of birth, citizenship, or both. Information on migrant-status stemming from citizenship has several shortcomings and is not a valid indicator of assessing migrant status. For example, the group of persons holding the national citizenship may be a jumble of the indigenous population, migrants, and their descendants. Country of birth can allow for identification of migrants, yet, persons born abroad of parents stemming from the EU country in question will be in the same category as more vulnerable migrants. Nevertheless, the ethnicity terminology, which refers to a group to which persons belong due to certain shared characteristics, including geographical origin, cultural traditions and languages, is not easily measured as it is imprecise and fluid [20]. From a practical perspective, a categorization stemming from a combination of both country of birth, country of birth of parents and citizenship will probably give the most non-biased identification of a migrant [21]; yet, it will not provide any solution to identifying ethnic minorities living in a country for generations. None of the registries carried information on self-assessed ethnicity e.g. stemming from a census which could encompass a subjective measure and furthermore, allowing identification of minority groups living in the country for generations [20]. This might be relevant in some of the EU countries with long migration history e.g. the UK [22] and for many other countries in the future.

Another striking feature is that no information on type of migration was available in the registries. This variable is important as it captures characteristics of the migration process (e.g. forced or voluntary migration) which are likely to have substantial impact on health needs and access issues [23,24] e.g. capability of adaptation and understanding of a new healthcare system. Also, duration of stay in the receiving country is of significance for use of healthcare services [25-27]; yet, this information can be obtained by linkage in some countries.

Finally, only six countries reported registries that contained information on socioeconomic factors which is relevant among others if one wishes to identify high risk groups. In the Scandinavian countries, availability of personal identification numbers allows for linkage of all national registries. Still, many EU countries are reluctant to implement the same identification system due to e.g. historical reasons and political and ethical concerns.

### Challenges of comparisons

The diversity of the available information on both healthcare utilization and migrant status and the non-existence of regional or national registry information in 16 EU countries makes comparisons across the EU difficult.

Firstly, the shortages on data availability on healthcare utilization limit the comparison possibilities. This is problematic as a defective picture of the subgroups' use of the various healthcare services might lead to wrong conclusions. Secondly, the groups of migrants differ from country to country, and the number of each migrant group might be very small in some countries. Several studies have demonstrated the great differences in health and health behaviour between the migrant groups [5,6,28] for which reason it is crucial to compare similar migrant groups across the different countries. Additionally, in the Belgium case, citizenship is based on three broad categories containing very heterogeneous groups which may lead to biased analyses. Since data need to be similar, including being based on the same categories of migrants defined in a similar way (see table 4), presently only limited comparisons across the EU countries can be carried out. Finally, when making comparisons, the complexicity of the various phenomenon (healthcare systems, migrants, social system etc.) of the countries needs to be taken into account as well as caution against inferring causality without the relevant evidence or proper understanding of the context and categories [29].

### Some recommendations

A sound scientific database on migrant-specific healthcare utilization is important for addressing problems in access to healthcare; to target initiatives; and to benchmark healthcare systems against each other in the EU. This requires implementation of registries in all EU countries in which indicators on healthcare utilization and migrant status are harmonized. By employing common definitions of migrants and common tools to collect data on migrants over time as well as on use of healthcare services, systematic documentation of and surveillance of health and health behaviour of migrants can be facilitated. Likewise, development of guidelines for comparisons, including analytical strategies which allow for adjustment of socio-economic factors, are essential.

## Conclusion

Results from this study underline the lack or shortage of data in many EU countries as well as the differences in the way migrants are identified. This impedes valid comparisons of healthcare utilization between migrants and the indigenous population across Europe based on existing registry data. Therefore, there is an urgent need to establish healthcare utilization registries, to expand existing registry information, and to adopt common, generally acceptable definitions of, and methods for identifying migrants in registries across the EU.

## Competing interests

The authors declare that they have no competing interests.

## Authors' contributions

SSN conceived of the study, participated in the design, collected the information on registries on healthcare utilization via the questionnaire, interpreted the results, and drafted the manuscript. AK conceived of the study, and participated in its design, interpreted the results and revised the manuscript. AR participated with providing supplementary data from a study that he conducted earlier and revised the manuscript. All authors read and approved the final manuscript.

## Pre-publication history

The pre-publication history for this paper can be accessed here:

http://www.biomedcentral.com/1472-6963/9/210/prepub
